# A β-Wrapin Targeting the N-Terminus of α-Synuclein Monomers Reduces Fibril-Induced Aggregation in Neurons

**DOI:** 10.3389/fnins.2021.696440

**Published:** 2021-07-13

**Authors:** Éva M. Szegő, Fabian Boß, Daniel Komnig, Charlott Gärtner, Lennart Höfs, Hamed Shaykhalishahi, Michael M. Wördehoff, Theodora Saridaki, Jörg B. Schulz, Wolfgang Hoyer, Björn H. Falkenburger

**Affiliations:** ^1^Department of Neurology, Technische Universität Dresden, Dresden, Germany; ^2^Department of Neurology, RWTH Aachen University, Aachen, Germany; ^3^Institut für Physikalische Biologie, Heinrich-Heine-Universität Düsseldorf, Düsseldorf, Germany; ^4^Institute of Biological Information Processing (IBI-7), Forschungszentrum Jülich GmbH, Jülich, Germany; ^5^JARA-Institute Molecular Neuroscience and Neuroimaging, Forschungszentrum Jülich GmbH and RWTH Aachen University, Aachen, Germany; ^6^Deutsches Zentrum für Neurodegenerative Erkrankungen, Dresden, Germany

**Keywords:** α-synuclein, pre-formed fibrils, protein aggregation, molecular chaperones, nanobodies

## Abstract

Reducing α-synuclein pathology constitutes a plausible strategy against Parkinson’s disease. As we recently demonstrated, the β-wrapin protein AS69 binds an N-terminal region in monomeric α-synuclein, interferes with fibril nucleation, and reduces α-synuclein aggregation *in vitro* and in a fruit fly model of α-synuclein toxicity. The aim of this study was to investigate whether AS69 also reduces α-synuclein pathology in mammalian neurons. To induce α-synuclein pathology, primary mouse neurons were exposed to pre-formed fibrils (PFF) of human α-synuclein. PFF were also injected into the striatum of A30P-α-synuclein transgenic mice. The extent of α-synuclein pathology was determined by phospho-α-synuclein staining and by Triton X-100 solubility. The degeneration of neuronal somata, dendrites, and axon terminals was determined by immunohistochemistry. AS69 and PFF were taken up by primary neurons. AS69 did not alter PFF uptake, but AS69 did reduce PFF-induced α-synuclein pathology. PFF injection into mouse striatum led to α-synuclein pathology and dystrophic neurites. Co-injection of AS69 abrogated PFF-induced pathology. AS69 also reduced the PFF-induced degeneration of dopaminergic axon terminals in the striatum and the degeneration of dopaminergic dendrites in the substantia nigra pars reticulata. AS69 reduced the activation of astroglia but not microglia in response to PFF injection. Collectively, AS69 reduced PFF-induced α-synuclein pathology and the associated neurodegeneration in primary neurons and in mouse brain. Our data therefore suggest that small proteins binding the N-terminus of α-synuclein monomers are promising strategies to modify disease progression in Parkinson’s disease.

## Background

In Parkinson’s disease (PD) and other synucleinopathies, aggregation and accumulation of α-synuclein (aSyn) is considered a central event. In PD and dementia with Lewy bodies (LBs), the major hallmarks of aSyn pathology are large inclusions in the neuronal soma (LB) and dystrophic, aSyn-containing neurites [Lewy neurites (LN)]. Reducing the extent of aSyn pathology thus represents an attractive neuroprotective strategy against synucleinopathies (Obeso et al., 2017).

Molecular chaperones can prevent protein aggregation (Muchowski and Wacker, 2005). Naturally occurring chaperones interact with aSyn *via* weak and transient interaction (Jia et al., 2019). The engineered β-wrapin AS69, in contrast, binds monomeric aSyn with high effectivity and high specificity (Mirecka et al., 2014). AS69 wraps around a sequence region of monomeric aSyn comprising residues 37–54 and stabilizes a β-hairpin conformation (Mirecka et al., 2014). AS69 therefore represents a new paradigm in amyloid inhibition. The aSyn N-terminal region is critical for aSyn aggregation (Mirecka et al., 2014; Shaykhalishahi et al., 2015; Doherty et al., 2020; Khammari et al., 2020). On a biophysical level, AS69 binding interferes with primary and secondary nucleation processes and inhibits the proliferation of aSyn fibrils (Agerschou et al., 2019). In HEK293T cells, AS69 reduces oligomerization and aggregation of aSyn; in a fruit fly model of A53T aSyn toxicity, AS69 reduces aggregation of aSyn in neurons and rescues the locomotor deficit resulting from neuronal aSyn expression (Agerschou et al., 2019).

So far, AS69 has not been studied in mammalian neurons. We therefore tested the effect of AS69 on aSyn pathology induced by pre-formed fibrils (PFF) in primary cortical neurons using a standard protocol (Volpicelli-Daley et al., 2014). In addition, we tested AS69 in the more complex biological environment of a mammalian brain using PFF injection in transgenic mice expressing human A30P-aSyn.

## Methods

The sources of chemicals are listed in Table 1. The source and concentration of antibodies are listed in Table 2. Supplementary Table 1 lists the composition of buffers, equipment, and software.

**TABLE 1:** Source of chemicals.

**Reagent**	**Source**	**ID**
Thioflavin T	Sigma-Aldrich	Cat# T3516
Poly-L-ornithine hydrobromide (0.1 mg/ml)	Sigma-Aldrich	Cat# 3655
LDH Cytotoxity Detection Kit	Roche	Cat# 11644793001
Bovine Serum Albumin (BSA)	Sigma-Aldrich	Cat# A2153
6× Loading Dye	Thermo Fisher	Cat# R0611
SuperSignal^TM^ West Femto Maximum Sensitivity Substrate	Thermo Fisher	Cat# 34096
Avidin-Biotin Complex from VECTASTAIN^®^ Elite ABC-HRP Kit (1:200)	Vector Laboratories	Cat# PK6100
SIGMAFAST^TM^ DAB with Metal Enhancer	Sigma-Aldrich	Cat# D0426
Entellan	Merck Millipore	Cat# 1079600500
Fluoromount-G	Southern Biotech	Cat# 0100-01
Hoechst 33342	Thermo Fisher	Cat# H3570
Novex^TM^ 4-20% Tris-Glycin Plus Gel	Thermo Fisher	Cat# NP0343

**TABLE 2:** Source and concentration of antibodies.

**Antibody**	**Source**	**ID**	**Dilution**
Mouse anti-α-Synuclein	BD Transduction Laboratories	Cat# 610787	1:2000
Goat anti-affibody	Abcam	Cat# ab50345	1:500
Rabbit anti-tubulin	Abcam	Cat# ab6046	1:2000
Rabbit anti-phospho-α-synuclein	Abcam	Cat# ab51253	1:500
Mouse anti-phospho-α-synuclein	Wako	Cat# 015-25191	1:1000
Rat monoclonal anti-human-α-synuclein (15G7)	Enzo Life Sciences	Cat# ALX-804-258-L001	1:500
Rabbit anti-MAP2	Merck Millipore	Cat# AB5622	1:1000
Rabbit anti-TH	Merck Millipore	Cat# AB152	1:1000
Chicken anti-GFAP	Abcam	Cat# ab4674	1:2000
Rabbit anti-Iba1	Wako	Cat# 019-19741	1:1000
Rabbit monoclonal rodent specific anti-α-synuclein (D37A6)	Cell Signaling	Cat# mAb4179	1:1000
Mouse monoclonal anti-aggregated-α-synuclein (5G4)	Merck Millipore	Cat# MABN389	1:1000
HRP-conjugated donkey anti-goat IgG	Jackson ImmunoResearch	Cat# 705-035-147	1:5000
HRP-conjugated donkey anti-mouse IgG	Jackson ImmunoResearch	Cat# 715-035-150	1:5000
HRP-conjugated donkey anti-rabbit IgG	Jackson ImmunoResearch	Cat# 711-035-152	1:5000
Biotinylated goat anti-rabbit IgG	Vector Laboratories	Cat# BA-1000	1:200
Alexa 405-conjugated donkey anti-rabbit	Jackson ImmunoResearch	Cat# 711-475-152	1:1000
Alexa 488-conjugated donkey anti-rat	Invitrogen	Cat# A21208	1:1000
Alexa 555-conjugated donkey anti-goat	Invitrogen	Cat# A21432	1:1000
Alexa 555-conjugated donkey anti-rabbit	Invitrogen	Cat# A31572	1:1000
Alexa 647-conjugated donkey anti-mouse	Invitrogen	Cat# A31571	1:1000
Alexa 488-conjugated goat anti-rabbit	Invitrogen	Cat# A-11008	1:1000
Alexa 488-conjugated goat anti-chicken	Invitrogen	Cat# A-11039	1:1000

### Recombinant Proteins and Atomic Force Microscopy

Human wild-type (WT) aSyn and AS69 were produced in bacteria and purified as previously described (Gauhar et al., 2014). PFF were generated using a standard protocol (Volpicelli-Daley et al., 2014) as previously described (Agerschou et al., 2019). Fibril formation was confirmed by Thioflavin T fluorescence and atomic force microscopy (AFM) (Agerschou et al., 2019).

Fibrils were analyzed by AFM. One microliter of sonicated PFF solution (5 mg/ml α-synuclein) was diluted with phosphate buffered saline solution (PBS) to 50 μl and adsorbed for 30 min onto a freshly cleaved mica surface followed by washing with milliQ water and drying with a gentle stream of N2 gas. Imaging was performed under air-dried conditions in intermittent contact mode in a JPK Nano Wizard II atomic force microscope using a silicon cantilever with a silicon tip (OMCL-AC160TS, Olympus) with a typical tip radius of 9 ± 2 nm, a force constant of 42 N/m, and a line rate of 0.5 Hz. The images were processed using JPK Data Processing software. Fibril lengths were determined using ImageJ software (Schindelin et al., 2012; Schneider et al., 2012) and the Ridge Detection plugin v1.4.0 (Steger, 1998). Supplementary Figure 1A shows a representative AFM image of our sonicated PFF, and Supplementary Figure 1B shows the size distribution of the PFF fragments.

### Animals and Surgery

C57BL6/J-Thy1-A30P-α-synuclein mice (Kahle et al., 2000) were bred as homozygous, housed, and handled in a pathogen-free animal facility at 20–24°C with a 12-h light/dark cycle and food and water *ad libitum*, in accordance with guidelines of the Federation for European Laboratory Animal Science Associations (FELASA). Breeding and surgery were approved by the local authorities (Landesamt für Natur, Umwelt und Verbraucherschutz Nordrhein-Westfalen, license numbers 84.02.04.2015.A027 and 84-02.04.2014.A321).

Mice (47–57 weeks old; males:females = 2:3 in PBS only, 2:4 in PFF only, 2:4 in PFF + ASS) were randomly assigned to one of the three experimental groups. The injected solution was prepared from frozen aliquots and sonicated on the day of the experiment. The final solutions contained (1) PBS only, (2) 1.4 mg/ml aSyn equivalent PFF, and (3) 1.4 mg/ml aSyn equivalent PFF + 98 nm. AS69. (98 μM AS69 is roughly equimolar to 1.4 mg/ml aSyn.).

Stereotaxic injection into the right striatum (AP: 1; ML: 1.5 relative to Bregma; DV: 1.55 from dura) and tissue preparations were performed as described earlier (Krenz et al., 2009), under ketamine (100 mg/kg)/xylazine (10 mg/kg) anesthesia (Guillen, 2012); 2.5 μl solution was injected with a flow rate of 0.2 μl/min. Surgeries and animal perfusions were performed between 8:00 and 16:00. After surgery, animals were kept in the original cage (3–5 mice per cage) and monitored every day for 10 days. Weight, wound healing, fell condition, and general behavior were scored according to the local Animal Welfare Authorities and the FELASA recommendations (Guillen, 2012). Mice were sacrificed 90 days later by an overdose of ketamine. Brains were fixed at 4°C (4% paraformaldehyde, 24 h) and cryoprotected (30% sucrose). Free-floating, 30-μm serial coronal sections were cut in a cryostat and were stored at −20°C until use. Nineteen mice started the experiment. Two animals died during the course of the experiment; the brains of these animals were not used for analysis. No power analysis was conducted to determine group size.

### Primary Neuronal Cultures

Primary neuronal cultures were prepared from 1- to 3-day-old C57BL6/J mouse pups (mixed sex, 4–6 pups/preparation). Dissociated neurons were plated onto poly-L-ornithine-coated glass coverslip (100,000 cells per well in 24-well dishes), and maintained in Neurobasal A medium [2% B27, 0.5 mM glutamax, antibiotics as previously (Szegő et al., 2019)]. One-third of the medium was changed on every third day, and from the second change on, no antibiotics were added. For image analysis, bovine serum albumin (BSA) (1 μg/ml, protein control), 50 nM AS69, PFF corresponding to 50 nM aSyn monomer, or the same concentration of PFF + 50 nM AS69 was added to neurons on day *in vitro* (DIV) 12. For detergent solubility fractionation, 150 nM AS69 and 150 nM aSyn were used. Neurons were analyzed on DIV 13 (24 h after adding PFF), DIV 15 (72 h after PFF), or DIV 22 (10 days after PFF). Experiments were repeated with three to four independent preparations (*n* = 3–4).

### Detergent-Solubility Fractionation

For detection of Triton X-100-insoluble proteins, neuronal cultures were lysed in buffer containing 1% Triton X-100 as previously described (Szegő et al., 2019). After centrifugation (14,000 × *g*, 30 min, 4°C), the supernatant was used as the Triton X-100 soluble fraction. The pellet (Triton X-100-insoluble fraction) was washed in ice-cold PBS, centrifuged, and re-dissolved with sonication (10 s) in 50 μl of buffer containing 2% SDS. Ten micrograms of the Triton X-100 soluble fraction or 10 μl from the Triton X-100-insoluble fraction was loaded onto a 4%–20% Tris/glycine SDS gel for Western blot analysis. aSyn, AS69, and tubulin were detected using an LAS-3000 Luminescent Image Analyzer with CCD camera. In the detergent-insoluble fraction, aSyn was not detected in the control conditions, “BSA only” and “AS69 only” (Figure 2D). The ratio of aSyn/tubulin is therefore presented as percent of the PFF-treated values (Figure 2E). In the detergent-soluble fraction (Supplementary Figure 2A), the ratio of aSyn/tubulin is reported as percent of the control condition BSA (Supplementary Figures 2B,C).

### Immunostaining–Brain Sections

To visualize the α-synuclein pathology, every sixth section of the striatum was used. After blocking endogenous peroxidase activity (0.3% H_2_O_2_), sections were first blocked (3% normal goat serum, 60 min), then incubated with a primary anti-phospho-aSyn antibody (overnight, 4°C, in blocking solution), followed by biotinylated secondary antibody and Avidin-Biotin Complex (30 min, 21°C each). Antibody labeling was visualized by 3,3′ diaminobenzidine (4 mg/ml). After mounting and dehydration in xylene, sections were coverslipped with Entellan.

To determine density of striatal dopaminergic axon terminals, three sections [0.26–0.98 mm to Bregma (Paxinos and Franklin, 2001)] per animal were stained for tyrosine hydroxylase (TH) as previously described (Komnig et al., 2016). In brief, after blocking, sections were incubated with TH antibody (overnight, 4°C) followed by secondary antibody (Alexa 488 conjugated goat anti-rabbit, 60 min). Sections were mounted with Fluoromount-G.

To determine the density of dopaminergic apical dendrites in the substantia nigra pars reticulata (SNr), every third section spanning the substantia nigra was stained for TH with DAB as described for the striatal sections.

To determine neuroinflammation, every sixth striatal sections was incubated with the astroglia marker glial fibrillary acidic protein (GFAP) and the microglia marker ionized calcium-binding adapter molecule 1 (Iba1) (overnight, 4°C). After incubation with fluorescently labeled secondary antibodies (Alexa 488-conjugated goat anti-chicken and Alexa 555-conjugated donkey anti-rabbit, 120 min), sections were counterstained with Hoechst and mounted with Fluoromount-G.

### Immunostaining–Primary Neurons

To determine aSyn uptake and pathology in primary neurons, cells were fixed 24 or 72 h after treatment and permeabilized (0.2% Triton X-100), unspecific sites were blocked (2% BSA), and cells were incubated in the presence of the following primary antibodies (4°C, overnight): phospho-aSyn, human-aSyn, affibody, and MAP2.

To address Triton X-100-insoluble aSyn pathology 10 days after seeding, neurons were fixed for 10 min (4% paraformaldehyde, 4% sucrose, and 1% Triton X-100), and washed with 0.1% Triton X-100. Neurons were stained with anti-MAP2, an antibody against aggregated aSyn, and an antibody recognizing mouse and rat aSyn, but not human aSyn (Villar-Piqué et al., 2016). After incubation with fluorescently labeled secondary antibodies, coverslips were mounted with Fluoromount G. To measure toxicity of compounds, neurons were fixed 10 days after treatment and stained for MAP2.

### Image Analyses–Brain Sections

Image analyses were carried out blinded for the experimental group by assigning random numbers to slides. aSyn pathology in the striatum was quantified in every third section spanning the entire striatum using stereological techniques and the optical fractionator method. In this method, phospho-aSyn positive somatic inclusions and dystrophic neurites (DNs) were counted manually (63× oil objective, Axio Imager 2 microscope, Carl Zeiss Vision) in counting frames presented by the software (StereoInvestigator, MicroBrightfield Bioscience, grid size: 200 μm × 200 μm, counting frame: 100 μm × 100 μm).

To quantify the density of striatal dopaminergic axon terminals (“fibers”), z-stack images were acquired (five planes, 1 μm apart, 60× oil objective, IX81S1F microscope, Olympus). TH-positive fibers were delineated from the maximal intensity projection (ImageJ, 1.47v) and expressed as percent area. Three sections per animal and five images per section were analyzed in a hierarchically nested design. A generalized linear mixed model (glm) was applied as noted in the *Statistical analysis and data visualization* section as previously described (Szegő et al., 2013).

For quantification of the density of TH-positive apical dendrites in the SNr, bright-field images were acquired (DAB staining) using a 20× objective (NA 0.8) with an Axio Imager 2 microscope (Zeiss). Area fraction of dendrites in the entire SNr was determined from a minimum of three sections per animal, and the ratio of ipsilateral and contralateral densities was analyzed.

For quantification of gliosis, fluorescent images were acquired (20× objective, Axio Imager 2 microscope, Zeiss). The area fraction of GFAP or Iba1 staining was determined from two sections per animal and from 10 images per section (ImageJ). Results were analyzed as described for TH-positive fibers. Fluorescent intensity of the GFAP signal was measured within the astroglia as follows using ImageJ. First, the image was duplicated, and one image was despeckled. The background was subtracted from the duplicate and then it was binarized and noise removal (despeckling) was applied. A mask was created from the binarized image. The mask was restored in the first image, and signal integrated density was measured within the mask. For quantification of microglia morphology, skeleton analysis was used (Young and Morrison, 2018). Briefly, individual glia cells were cropped from the binarized images (see above), and after noise removal (despeckling), using the close function (connecting two pixels that are separated by up to two pixels) and removing outliers (pixel radius of 2, threshold of 50), cells were skeletonized. Total branch length per cell was calculated by summing up the length of branches over a cutoff value of 2.

### Image Analyses–Primary Neurons

**FIGURE 1:**
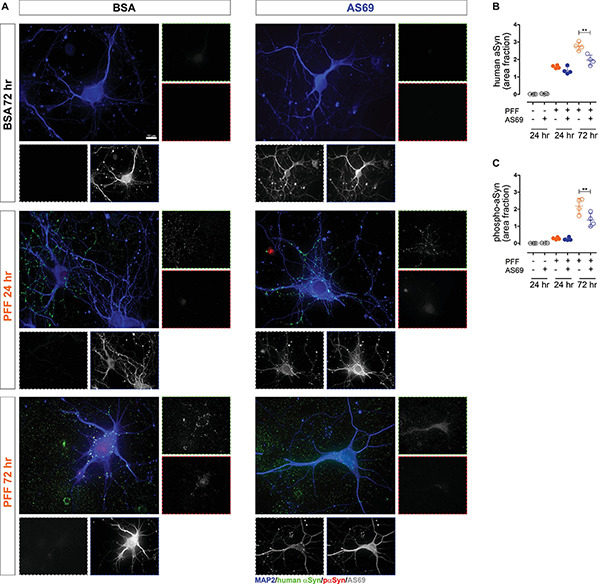
AS69 does not affect PFF uptake, but reduces early pathology. **(A)** Representative images of primary neurons acquired 24 or 72 h after treatment with either PFF or BSA, and in addition either AS69 or BSA. Neurons were stained for AS69 (inset with white border, not included in merged image), phospho-aSyn (red border inset and red channel in merged image), human aSyn (green border inset and green channel), and neuronal markers [microtubule-associated protein 2 (MAP2) + βIII-tubulin, blue border inset and blue channel]. Scale bar: 10 μm. **(B)** Quantification of the area positive for human aSyn within the area delineated by the neuronal markers in neurons as depicted in panel **(A)** (*n* = 4 independent preparations, ≥10 neurons per experimental group, *p* = 0.007 for PFF + BSA vs. PFF + AS69 at 72 h, two-way ANOVA followed by Bonferroni *post hoc* test). **(C)** Quantification of phospho-aSyn within the same neurons as in panel **(B)** (*p* = 0.009 for PFF + BSA vs. PFF + AS69 at 72 h, two-way ANOVA followed by Bonferroni *post hoc* test). All markers represent the mean of all technical replicates of one individual preparation; lines represent mean ± SD of independent preparations. **p < 0.01.

**FIGURE 2:**
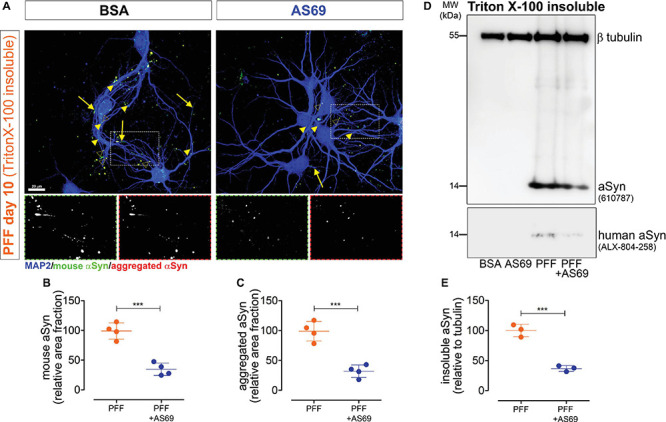
AS69 inhibits PFF-induced aggregation of endogenous aSyn monomers in primary neurons. **(A)** Representative images of primary neurons acquired 10 days after seeding with PFF + BSA or PFF + AS69. Neurons were incubated with 1% Triton X-100 before staining to remove Triton X-100 soluble aSyn and stain only Triton X-100-insoluble aSyn. Channels: mouse aSyn (green), aggregated aSyn (red), and neuronal markers (blue). Arrowheads: dot-like aSyn inclusions; open arrowheads: bigger, round inclusions; arrow: fibrillary inclusions. Scale bar: 20 μm. **(B)** Quantification of endogenous mouse aSyn in primary neurons treated as in panel **(A)**, normalized to the “PFF only” condition (*n* = 4 independent preparations, ≥10 neurons per experimental group, *p* = 0.0003, *t*-test). **(C)** Quantification of aggregated aSyn staining in the same neurons as in panel **(A)**, normalized to the “PFF only” condition (*p* = 0.0004, *t*-test). **(D)** Immunoblot of the Triton X-100-insoluble fraction of primary neuron lysates obtained 10 days after PFF treatment. Blots were incubated with an antibody detecting both mouse and human aSyn (BD 610787) and with an antibody against βIII-tubulin as loading control. The immunoblot of the Triton X-100 soluble fraction of the same lysate and its quantification are in Supplementary Figure 2. **(E)** Quantification of the 14 kDa band of *n* = 3 independent blots as in panel **(D)**, showing intensity of the aSyn band relative to the βIII-tubulin band with the “PFF-only” condition set to 100% (*p* = 0.0006, *t*-test). All markers represent the mean of all technical replicates of one individual preparation; lines represent mean ± SD of independent preparations. ***p < 0.001.

To measure aSyn uptake and pathology in neurons, a minimum of 10 neurons were imaged randomly for each experiment and experimental group using a 100× oil objective on a Zeiss Axio Imager 2 microscope and constant exposure times for each staining across all experimental groups. To outline neurons, a mask was created based on the MAP2 channel. In this mask, the area fraction of staining for (a) phospho-aSyn, (b) human aSyn, (c) mouse aSyn, or (d) aggregated aSyn was determined, using ImageJ. Images are pseudo colored for better visualization. For phospho-aSyn and human aSyn, values are reported as “raw” area fraction (Figures 1B,C). For the staining after incubation with 1% Triton X-100 (Figures 2B,C), area fractions are normalized to values of only PFF-treated neurons.

Neuronal integrity was quantified by measuring the area of MAP2-positive signal from the primary cultures. For that, images were acquired with a 20× objective, background was subtracted, and images were despeckled and binarized by thresholding. Outliers (pixel radius of 2, threshold of 50) were removed from the binary image. The area fraction was determined from the binary image.

### LDH Assay

To determine the toxic effects of aSyn PFF on primary neuronal cultures, the concentration of extracellular LDH was measured in the medium 24 h after the last medium change using the Cytotoxicity Detection Kit according to the manufacturer’s protocol. Briefly, for each independent preparation, three to four technical replicates were measured and the results were averaged. Absorbances were measured at 492 nm; reference wavelength was 620 nm. Background (medium) absorbance was subtracted from all values, and values were normalized to control cells (non-treated) and to maximal lysed cells (treated with 2% Triton X-100), according to the protocol.

### Statistical Analysis and Data Visualization

“*n*” was set to the number of individual preparations for cell culture experiments or to the number of animals for the animal experiments. Sample sizes are based on previous experience and not on a calculation performed prior to the experiment. Data are presented as markers for each experiment/animal and summarized as mean ± standard deviation of these markers. Data normality was tested by the Shapiro–Wilk test (R). No test for outliers was performed and no data points were removed. To compare experimental groups, we used *t*-test, one-way or two-way ANOVA in GraphPad Prism 5 (Version 5.01), or a linear mixed effect model that allows both fixed and random effects (animal and image in the hierarchically nested design) as previously described (Szegő et al., 2013) in R (version 2.8.0).

*p* < 0.05 was considered statistically significant. *p*-values are depicted on the graphs as ^∗^*p* < 0.05, ^∗∗^*p* < 0.01, and ^∗∗∗^*p* < 0.001, and exact *p*-values and *r*^2^ values are noted in the figure legends.

## Results

### AS69 Decreases Intracellular aSyn Pathology in Primary Neurons

AS69 is a small (15 kDa) protein that interferes with aSyn aggregation in a substoichiometric way (Mirecka et al., 2014; Agerschou et al., 2019). Its mechanism includes the inhibition of secondary nucleation, which is a critical part of the prion-like behavior of aSyn (Törnquist et al., 2018). Here, we modeled secondary nucleation in primary neurons by applying PFF prepared from human WT aSyn. In neurons exposed to PFF, human aSyn staining was detected intracellularly 24 h after adding PFF (Figures 1A,B), confirming that primary neurons take up PFF–as demonstrated previously by others (Volpicelli-Daley et al., 2014). The extent of early PFF-induced aSyn pathology was determined by staining for phospho-aSyn 24 and 72 h after seeding (Figures 1A,C). Phosphorylation of aSyn is widely used to quantify seeded aSyn pathology (Mahul-Mellier et al., 2020; Weston et al., 2021).

We demonstrated previously that AS69 does not dissociate aSyn fibrils (Gauhar et al., 2014; Mirecka et al., 2014; Agerschou et al., 2019) and therefore added AS69 together with the PFF; BSA was used as negative control. In neurons incubated with AS69 but not PFF, we observed intracellular AS69 staining (Figure 1A, BSA + AS69 group), confirming that neurons can take up AS69 independently of PFF. AS69 did not alter the amount of intracellular human aSyn at 24 h (Figure 1B), indicating that AS69 does not affect aSyn uptake–in agreement with our previous finding that AS69 does not directly affect aSyn fibrils.

When neurons were exposed to PFF with AS69, the extent of phospho-aSyn pathology after 72 h was reduced by 37% compared to PFF alone (Figure 1C), consistent with our previous finding that AS69 reduces secondary nucleation *in vitro* (Agerschou et al., 2019). The area positive for human aSyn at 72 h was also reduced by 29% by AS69 (Figure 1B).

In order to investigate the effect of AS69 on PFF-induced aSyn pathology over a longer time period, we measured the fraction of detergent-insoluble aSyn 10 days after PFF seeding, as described earlier by others (Volpicelli-Daley et al., 2014). Detergent-insoluble aSyn is considered a pathological form (Klucken et al., 2006). Thus, PFF-treated neurons were incubated with 1% Triton X-100 after fixation to remove soluble aSyn. Neurons were then stained for endogenous mouse aSyn and aggregated aSyn (Figure 2A). We observed aSyn-positive puncta (arrowheads), bigger, roundish aggregates (open arrowheads), and longer aggregates (arrows). Quantification of Triton X-100-insoluble mouse aSyn (green in Figure 2A) and aggregated aSyn (red in Figure 2A) showed that AS69 treatment reduced the amount of aSyn aggregates after PFF seeding by 65% and 67% (Figures 2B,C).

In addition, we performed detergent solubility fractionation of neuronal protein lysates obtained 10 days after adding PFF. Primary neurons exposed to PFF showed a strong accumulation of aSyn in the detergent-insoluble fraction (Figure 2D). AS69 reduced the amount of aSyn in the detergent-insoluble fraction by 63% (Figure 2E). The amount of aSyn in the soluble fraction was not altered (Supplementary Figure 2).

### AS69 Decreases aSyn-Induced Degeneration in Primary Neurons

**FIGURE 3:**
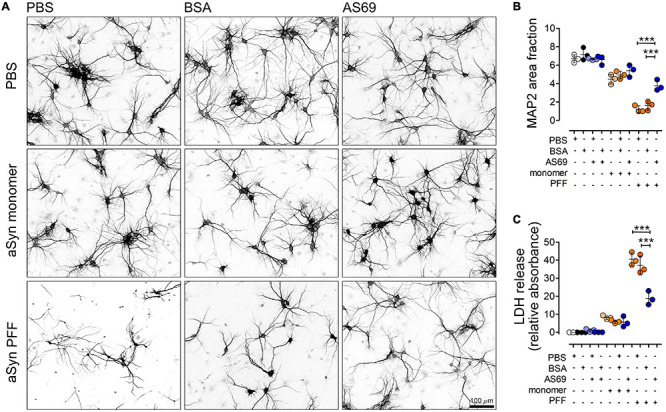
AS69 decreases aSyn-induced degeneration in primary neurons. **(A)** Representative images of primary neurons stained for MAP2 10 days after treatments as indicated. Scale bar: 100 μm. **(B)** Quantification of the area positive for MAP2 in neurons as depicted in panel **(A)** (*n* = 3 independent preparations, ≥10 image fields per experiment/group). Two-way ANOVA showed positive interaction (*p* = 0.004) (*p*-values of Bonferroni *post hoc* tests depicted). **(C)** Quantification of LDH release in primary neuronal cultures with groups as in panel **(A)**. Two-way ANOVA showed positive interaction (*p* = 0.004) (*p*-values of Bonferroni *post hoc* tests depicted). All markers represent the mean of all technical replicates of one individual preparation; lines represent mean ± SD of *n* = 3 independent preparations. ***p < 0.001.

In order to determine the functional relevance of the AS69 effects in primary neurons, we quantified the area covered by the MAP2-positive neuropil (Figures 3A,B) and measured cell death using the LDH assay (Figure 3C). aSyn monomers slightly decreased neuronal integrity, but to a much lesser extent than observed for PFF. PFF decreased the extent of neuronal processes substantially (Figures 3A,B) and induced cell death (Figure 3C). AS69 significantly reduced the extent of PFF-induced neuronal damage whereas BSA did not (Figures 3B,C).

### AS69 Reduces PFF-Induced α-Synuclein Pathology *in vivo*

**FIGURE 4:**
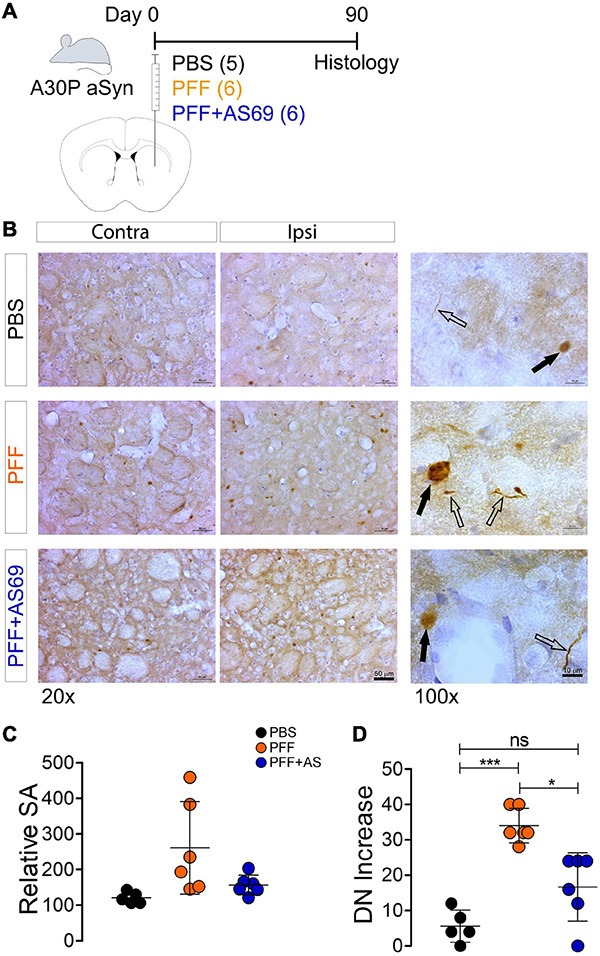
AS69 inhibits PFF-induced aSyn-pathology in mouse brain. **(A)** Injection scheme: Transgenic mice expressing human A30P-aSyn received injections into the striatum of either PBS as control, PFF prepared from human WT aSyn, or PFF + AS69. aSyn pathology was analyzed 90 days later. Nineteen mice started the experiment. Two animals died during the course of the experiment; brains of these animals were not used for analysis. **(B)** Representative, low-magnification (20×) and high-magnification (100×) images of striatal sections stained for phospho-aSyn. Dystrophic neurites (DN, open arrows) and somatic accumulations (SA, black arrows) are indicated. Scale bars: 50 μm (20×) and 10 μm (100×). **(C)** Quantification of the number of SA in the injected striatum normalized by the non-injected hemisphere to control for variance between animals (100 × ipsilateral/contralateral). Differences between groups were not statistically significant (one-way ANOVA followed by Bonferroni *post hoc* test). **(D)** Number of DN per section in the injected striatum normalized by the non-injected hemisphere (ipsilateral–contralateral; *p* = 0.0007 for PBS vs. PFF; *p* = 0.004 for PFF vs. PFF + AS69, one-way ANOVA followed by Bonferroni *post hoc* test). Absolute values for panels **(C,D)** are depicted in Supplementary Figures 3A,B. Markers represent values for individual animals; lines represent mean ± SD of these values, *n* = 5 (PBS) or 6 (PFF, PFF + AS69) animals. ns: not significant; *p < 0.05; ***p < 0.001.

Since AS69 reduced aSyn pathology in primary neurons, we next asked whether AS69 can also reduce PFF-induced aSyn pathology in mice. PFF were injected into the striatum of 47- to 57-week-old mice with neuronal expression of human A30P-aSyn. We used aSyn transgenic mice because the higher concentration of aSyn neurons in these mice facilitates aSyn aggregation, and because the human isoform expressed from the transgene may circumvent the species barrier between PFF prepared from human aSyn and the endogenous mouse aSyn observed in some studies (Luk et al., 2016). A30P-aSyn transgenic mice have been used for PFF-based seeding less frequently than transgenic mice with other aSyn variants. We used this line because we have worked with it extensively in the past (Rathke-Hartlieb et al., 2001; Krenz et al., 2009; Szegő et al., 2012). Mouse brains were analyzed 90 days after the injection (Figure 4A). To reveal PFF-induced striatal aSyn pathology, we visualized phospho-aSyn positive structures (Figure 4B) and discriminated two phenotypes: (i) somatic accumulations of phospho-aSyn (“SA,” solid arrows in Figure 4B, 100× images) and (ii) dystrophic, phospho-aSyn positive neurites [“DN,” open arrows in Figure 4B 100× images]. These changes are reminiscent of the somatic LB and LN in human brain.

Even in vehicle-injected mice, we saw a relevant number of SA (Figures 4B,C) and DN (Figures 4B,D), as demonstrated previously for A30P-aSyn mice (Schell et al., 2009; Fagerqvist et al., 2013). The injection of PFF induced the formation of additional SA and DN; the number of SA and DN was significantly higher in the injected hemisphere than in the non-injected hemisphere (Supplementary Figures 3A,B). To account for variability between animals and compare between treatment groups, baseline was subsequently defined as the non-injected hemisphere of the same animal and numbers normalized to this baseline. We observed a significant difference between PFF-injected animals and PBS-injected controls for the number of DN in the striatum (Figure 4D). The difference was not statistically significant for SA (Figure 4C).

Co-injection of AS69 decreased PFF-induced formation of DN by 49% (Figure 4D, values for individual hemispheres are in Supplementary Figure 3). These findings are consistent with our results in cultured neurons (Figures 1–3).

### AS69 Reduces Degeneration of Dopaminergic Axon Terminals and Dendrites

**FIGURE 5:**
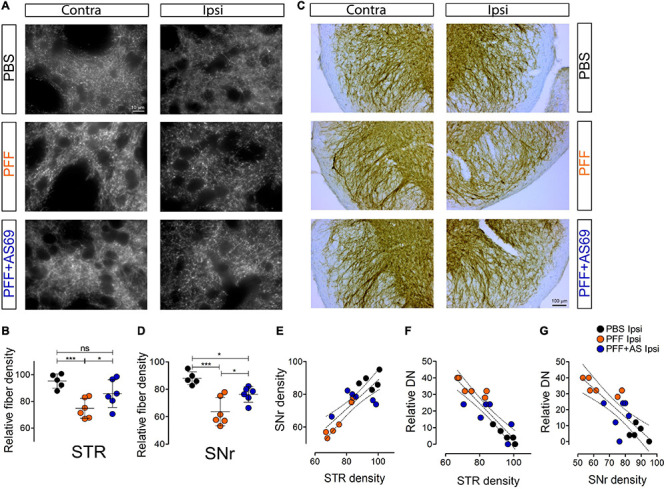
AS69 decreases PFF-induced degeneration of dopaminergic fibers. **(A)** Representative images of striatal sections ipsilateral and contralateral to the PFF injection stained for dopaminergic (TH positive) fibers 90 days after PFF injection. Scale bar: 10 μm. **(B)** Relative density of striatal dopaminergic fibers in the striatum (STR), 100 × ipsilateral/contralateral hemisphere, *p* = 0.0009 for PBS vs. PFF; *p* = 0.041 for PFF vs. PFF + AS69, linear mixed model. Absolute values are in Supplementary Figure 3C. **(C)** Representative images of TH-positive dendrites in the substantia nigra pars reticulata (SNr) 90 days after PFF injection. Scale bar: 100 μm. **(D)** Relative density of dopaminergic dendrites in the SNr, 100 × ipsilateral/contralateral hemisphere, *p* = 0.0008 for PBS vs. PFF; *p* = 0.012 for PFF vs. PFF + AS69, linear mixed model. **(E)** Linear regression of dopaminergic axon terminals in STR (from panel **B**) vs. dopaminergic dendrites in SNr (from panel **D**); *p* = 0.00067; *r*^2^ = 0.6843. **(F)** Linear regression of dopaminergic fibers in STR (from panel **B**) vs. DN (from Figure 2D); *p* = 0.00005; *r*^2^ = 0.8063. **(G)** Linear regression of dopaminergic dendrites in SNr (from panel **D**) vs. DN (from Figure 2D); *p* = 0.00072; *r*^2^ = 0.6535. In panels **(B,D–G)**, markers represent individual animals. In B and D, lines represent mean ± SD of these values. In panels **(E–G)**, dotted lines represent linear regression and 95% confidence interval. *n* = 5 (PBS) or 6 (PFF, PFF + AS69) animals. ns, not significant; *p < 0.05; ***p < 0.001.

A30P-aSyn transgenic mice do not show spontaneous degeneration of dopaminergic neurons in the substantia nigra or degeneration of their axon terminals in the striatum (Rathke-Hartlieb et al., 2001). Similarly, injection of PBS did not reduce the density of striatal dopaminergic axon terminals (Figures 5A,B), or the density of dopaminergic apical dendrites that extend from the SNc into the SNr (Figures 5C,D). Injection of PFF, in contrast, reduced the density of striatal dopaminergic axon terminals by 22% (Figures 5A,B) and the density of dopaminergic dendrites in the SNr by 28% (Figures 5C,D). This is consistent with previous findings in mice transgenic for A53T aSyn (Luk et al., 2012b).

Co-injection of AS69 abrogated the PFF-induced degeneration of dopaminergic axon terminals: The relative density was not significantly different from PBS-injected mice, and it was significantly higher than in PFF-injected animals (54% rescue; Figures 5A,B). Similarly, co-injection of AS69 abrogated the PFF-induced loss of dopaminergic dendrites in the SNr (51% rescue; Figures 5C,D). Across treatment groups, the density of dopaminergic dendrites in the SNr correlated strongly with the density of dopaminergic axon terminals in the striatum (Figure 5E; *r*^2^ = 0.6843; *p* = 0.00067), indicating that aSyn pathology affects the entire neuron.

Although it is widely accepted that aSyn pathology is a major hallmark of PD and a driver of neurodegeneration, it is still a matter of debate which type of aSyn inclusions correlates best with neurotoxicity and functional impairment (Schulz-Schaeffer, 2012). We therefore correlated on an animal basis the extent of SA and DN pathology with the density of dopaminergic fibers. We observed a strong and significant correlation between DN pathology and the reduction in dopaminergic fibers in the striatum (*r*^2^ = 0.8063; *p* = 0.00005; Figure 5F), and also with the reduction of dopaminergic dendrites in the SNr (Figure 5G, *r*^2^ = 0.6535, *p* = 0.00007). The extent of SA pathology, in contrast, did not correlate significantly with dopaminergic fiber loss in the striatum (*r*^2^ = 0.0335; *p* = 0.5067; Supplementary Figure 4A) or with the loss of dopaminergic dendrites in the SNr (Supplementary Figure 4B, *r*^2^ = 0.04519, *p* = 0.4127). This difference suggests that, in our model, neuritic aSyn pathology is more closely linked to the degeneration of dopaminergic axon terminals than the presence of SA.

### AS69 Reduces Activation of Astroglia by PFF

**FIGURE 6:**
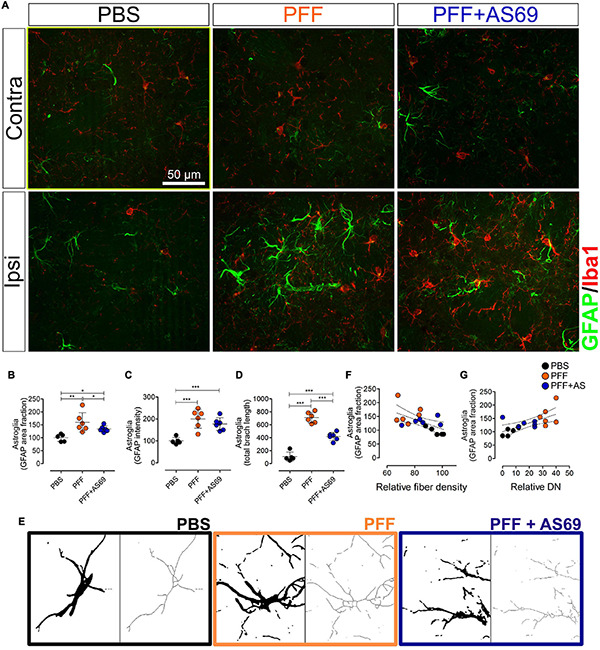
AS69 decreases PFF-induced astrogliosis. **(A)** Representative images of striatal sections ipsilateral and contralateral to the PFF injection stained for astroglia (GFAP, green) and microglia (Iba1, red) 90 days after PFF injection. Scale bar: 50 μm. **(B–D)** Astroglia reaction expressed as GFAP-positive area fraction **(B)**, GFAP staining intensity **(C)**, and skeleton branch length **(D)** in the injected hemisphere relative to the contralateral hemisphere with the PBS-injected group set to 100%. *p*-values from one-way ANOVA followed by Bonferroni *post hoc* test. Absolute values for individual hemispheres are in Supplementary Figures 3D–F. **(E)** Representative images of individual astroglia after binarization (left panels) and after skeletonization (right panels) as used for panel **(D)**. **(F)** Linear regression of astroglia activation (from panel **B**) vs. density of dopaminergic fibers in the striatum (from Figure 5B), *p* = 0.0159; *r*^2^ = 0.3298. **(G)** Linear regression of astroglia activation (from panel **B**) vs. DN in the striatum (from Figure 3D), *p* = 0.0046; *r*^2^ = 0.4238. In panels **(B–G)**, markers represent individual animals. In panels **(B–D)**, lines represent mean ± SD. In panels **(F,G)**, dotted lines represent linear regression and 95% confidence interval. *n* = 5 (PBS) or 6 (PFF, PFF + AS69) animals. *p < 0.05; **p < 0.01; ***p < 0.001.

Finally, we measured the glial response to PFF injection in the striatum by measuring the area positive for the astroglia marker GFAP (Figure 6A). PFF injection increased the area positive for GFAP (160% of PBS control; Figures 6A,B), suggesting activation of astroglia. PFF injection also increased the intensity of GFAP staining (Figure 6C) and the morphology of GFAP-positive cells (branch length per cell, Figures 6D,E). Injection of PBS alone did not produce a significant glial activation (Figures 6A–E), confirming that that the observed effects can be attributed to the PFF. AS69 reduced activation of astroglia by 43% (Figures 6A–D). The extent of astroglia activation correlated inversely with the density of dopaminergic fibers (*r*^2^ = 0.3298; *p* = 0.0159; Figure 6F) and positively with the relative density of DN (*r*^2^ = 0.4238; *p* = 0.046; Figure 6G). This correlation can best be explained by astroglia activation downstream of neuronal damage. The correlation of astroglia activation with the density of SA was not statistically significant (Supplementary Figure 4C).

In addition, we determined the response of microglia by measuring the area positive for Iba1 (Figure 6A). PFF injection induced activation of microglia (110% of PBS control, Supplementary Figure 4E), consistent with the finding by others that microglia plays an active role in the PFF-induced degeneration (Duffy et al., 2018; Earls et al., 2019). Microglia activation was not affected by AS69 (Figure 6A and Supplementary Figure 4E).

## Discussion

**FIGURE 7:**
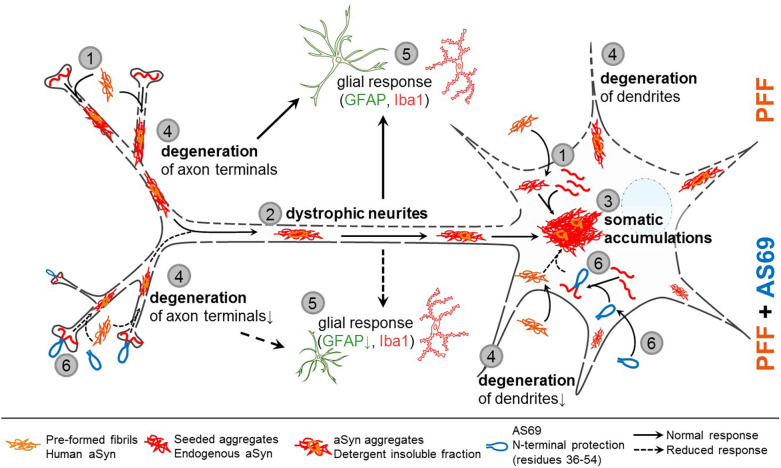
Proposed model of aSyn pathology and AS69 protection. **(1)** aSyn pre-formed fibrils (PFF) are taken up by neurons and accelerate aggregation of aSyn monomers into detergent-insoluble aggregates (see Figures 1D–G). **(2)** Phospho-aSyn positive aggregates accumulate in dystrophic neurites reminiscent of Lewy-neurites and to a lesser extent in somatic inclusions reminiscent of Lewy bodies (see Figure 2). **(3)** Neuritic aSyn aggregates are retrogradely transported to the neuronal soma, where they are collected into somatic accumulations. **(4)** aSyn pathology causes degeneration of dopaminergic axon terminals in the striatum and degeneration of dopaminergic dendrites in the SNr (see Figure 3). **(5)** Dystrophic neurites and their degeneration leads to a glial response (see Figure 4). **(6)** AS69 is taken up by neurons, binds to N-terminal residues of the aSyn monomer, and through this mechanism reduces nucleation of aSyn aggregates (see Figures 1C,E,G), dystrophic neurites (see Figure 2D), degeneration of axon terminals and dendrites (see Figures 3B,D) and the astroglial response (see Figure 4C).

Here, we investigated the effect of AS69, which binds the aSyn monomer with high affinity, in PFF-based models of secondary nucleation. In primary neurons, AS69 did not alter PFF uptake, but reduced aggregation of endogenous mouse aSyn after seeding with PFF. In mouse brain, AS69 ameliorated PFF-induced striatal aSyn pathology. AS69 also abrogated the degeneration of striatal dopaminergic axon terminals and the reduction of dopaminergic dendrites in the SNr, demonstrating the functional relevance of the aggregate reduction (summarized in Figure 7).

In primary neurons exposed to PFF, staining for human aSyn was already observed after 24 h whereas the extent of phospho-aSyn staining increased mainly between 24 and 72 h, consistent with previous findings that seeding of aSyn pathology by extracellular PFF takes time (Flavin et al., 2017; Rodriguez et al., 2018). During this time, PFF exit from endosomes, interact with endogenous aSyn, and seed the pathology visualized by the phospho-aSyn staining (Karpowicz et al., 2017; Wu et al., 2019). AS69 reduced the extent of phospho-aSyn pathology after 72 h (Figure 1C) and the extent of Triton X-100-insoluble aSyn after 10 days (Figure 2), indicating that AS69 reduces aSyn pathology. Staining for mouse aSyn showed Triton X-100-insoluble aggregates after PFF treatment (Figure 2B), confirming that the PFF made from human aSyn seeded aggregation of endogenous aSyn (Karpowicz et al., 2017; Wu et al., 2019). We used an antibody against “aggregated” aSyn, and conformation-specific antibodies have their limitations (Kumar et al., 2020). Yet, the strong co-localization with mouse aSyn staining after 1% Triton X-100 treatment (Figure 2A) suggests that the staining for aggregated aSyn in this paradigm represents for the most part Triton X-100-insoluble aSyn. Interestingly, AS69 also reduced the area positive for human aSyn after 72 h (Figure 1B). Therefore, the reduced extent of aSyn pathology observed with AS69 could alleviate the known inhibition of autophagy by aSyn pathology (Mazzulli et al., 2016; Hoffmann et al., 2019) and facilitate clearance of (human) aSyn. Improved clearance of human aSyn could also result from improved cellular health as suggested by the denser neuropil and the reduced LDH release in the presence of AS69 (Figure 3).

Despite the abundance of aSyn pathology (Figures 4B–D), nigral dopaminergic neurons and their striatal axon terminals are preserved in the A30P-aSyn transgenic mice used in this study (Figures 5A–D; Rathke-Hartlieb et al., 2001). In aSyn transgenic mice and in PFF-based models, pathology originates in the presynaptic compartment and propagates retrogradely to the cell body (Schaser et al., 2020). The presence of SA in the A30P-aSyn transgenic mice (Figure 4C) therefore suggests that retrograde transport to assemble aSyn aggregates in perinuclear accumulations is preserved in A30P-aSyn mice. PFF injection triggered a robust increase in aSyn pathology (Figures 4C,D), consistent with previous work using PFF in other aSyn transgenic mice (Luk et al., 2012a; Zhang et al., 2019). The PFF-induced increase was particularly strong for DN (Figure 4D). The predominant formation of DN in our paradigm can be explained: (1) by a very rapid formation of aSyn aggregates when PFF “seeds” are added to the high concentration of aSyn in neurons resulting from the combined expression of endogenous aSyn and the transgene; and (2) by the known inhibition of retrograde axonal transport by aSyn (Chung et al., 2009; Koch et al., 2015; Prots et al., 2018). In previous studies with PFF, SA-like aSyn pathology was mainly observed 4–6 months after PFF injection (Luk et al., 2012a, b; Osterberg et al., 2015; Zhang et al., 2019), thus later than our 3-month time point (Figure 4A). Consistent with the concept of retrograde progression noted above, we therefore assume that the abundance of SA would be more pronounced at a later time point. In addition, a slightly different phenotype in our model could be explained by the fact that 12-month-old mice were used in our experiments whereas most previous studies used younger animals. In our paradigm, the tight correlation between DN and (i) degeneration of dopaminergic axon terminals in the striatum (Figure 5F), (ii) degeneration of dopaminergic dendrites in the SNr (Figure 5G), and (iii) astrogliosis (Figure 6G) suggests that the DN pathology is responsible for these observed effects. The functional importance of aSyn pathology in neuronal processes is consistent with the finding of presynaptic aSyn microaggregates in the absence of somatic inclusions (Spinelli et al., 2014), earlier aSyn-induced oxidative stress in the synaptic terminals than in the soma (Szegő et al., 2019; Schaser et al., 2020), and the observation that presynaptic aSyn is a primary target for phosphorylation (Weston et al., 2021). As noted above, however, neuritic aSyn pathology often precedes somatic pathology, and cortical neurons with aSyn inclusions degenerate over time (Osterberg et al., 2015).

AS69 reduced PFF-induced DN formation (Figure 3D), and we propose that this underlies the protection of striatal axon terminals in the striatum (Figure 5B), dopaminergic dendrites in the SNr (Figure 5D), and the reduced astrogliosis (Figures 6B–D), consistent with our findings in primary neurons. The pathway of aSyn pathology and the proposed mechanism of AS69 action are summarized in Figure 7. We acknowledge that the reduction of PFF-induced aSyn pathology by AS69 was incomplete. The incomplete rescue might result from degradation of AS69 over time while the aSyn pathology increases (Patterson et al., 2019). In addition, due to the high concentration of human A30P-aSyn resulting from the transgene, aSyn fibrils–after PFF seeding–might grow mainly by fibril elongation, bypassing the need for fibril amplification by secondary nucleation. It will be important to test in future studies whether the effect of AS69 increases with repeated administration. From a translational standpoint, slowing down disease progression by 50% would already constitute a clinically meaningful effect in PD patients.

Limitations of this study include the use of transgenic mouse lines expressing human A30P-aSyn and the injection of PFF into the striatum. Both the distribution of aSyn pathology throughout the nervous system and the precise composition of the aSyn aggregates differ from PD patients. Also, we did not investigate the effects of AS69 on the propagation of aSyn pathology from the enteric nervous system to the central nervous system (Borghammer and Van Den Berge, 2019; Van Den Berge et al., 2019; Ferreira et al., 2021) that likely occurs in PD patients.

The N-terminal aSyn residues 35–43 constitute one of the hotspots controlling aSyn aggregation (Doherty et al., 2020; Khammari et al., 2020). They are targeted not only by AS69 but also by some of the aSyn antibodies tested for application in PD patients. Our findings about AS69 therefore indicate that molecules binding residues 35–43 of the aSyn monomer are promising candidates against PD.

## Disclosures

ÉS, FB, DK, CG, LH, HS, MW, and TS have no disclosure. JS has received payment from Biogen and grants from Deutsche Forschungsgemeinschaft, the Christina Foundation, and Pfizer. WH has received research support from the European Research Council. BF has received payment for talks from UCB and a grant from Deutsche Forschungsgemeinschaft.

## Data Availability Statement

The original contributions presented in the study are included in the article/Supplementary Material, further inquiries can be directed to the corresponding author/s.

## Ethics Statement

The animal study was reviewed and approved by Landesamt für Natur, Umwelt und Verbraucherschutz Nordrhein-Westfalen, license numbers 84.02.04.2015.A027 and 84-02.04.2014.A321.

## Author Contributions

ÉS, JS, WH, and BF conceived the research. ÉS, FB, DK, CG, LH, HS, MW, and TS performed the research. ÉS, WH, and BF wrote the manuscript. All authors contributed and approved the manuscript.

## Conflict of Interest

The authors declare that the research was conducted in the absence of any commercial or financial relationships that could be construed as a potential conflict of interest.

## Supplementary Material

Supplementary Figure 1 Characterization of sonicated PFFs by atomic force microscopy (AFM). **(A)** AFM amplitude image of sonicated PFFs. **(B)** Violin plot of PFF lengths determined AFM. The white dot at 41 μm represents the median. The thick gray bar represents the interquartile range, the thin gray line represents the rest of the distribution, except for points that are determined to be outliers using a method that is a function of the interquartile range.

Supplementary Figure 2 **(A)** Representative immunoblot showing the Triton X-100 soluble fraction of primary neuron lysates obtained 10 days after PFF treatment. Blots were first incubated with an antibody detecting only rodent aSyn (D37A6, upper image), then with an antibody detecting both rodent and human aSyn (BD610787, middle image), finally βIII-tubulin was detected as loading control (lower image). **(B)** Quantification of the rodent aSyn signal at the 14 kDa band of *n* = 3 independent blots as in panel **(A)**, showing intensity of the aSyn band relative to the βIII-tubulin band with the signal in the BSA-treated condition set to 100%. **(C)** Quantification of the total aSyn signal at the 14 kDa band of *n* = 3 independent blots as in panel **(A)**, showing intensity of the aSyn band relative to the βIII-tubulin band with the signal in the BSA-treated condition set to 100%.

Supplementary Figure 3 **(A,B)** DN and SA in the striatum in absolute numbers for each hemisphere, related to Figures 4C,D. **(C)** Density of dopaminergic axon terminals in the striatum in absolute numbers for each hemisphere, related to Figure 5B. **(D–F)** GFAP area fraction **(D)**, GFAP staining intensity **(E)** and total branch length after skeletonization of GFAP positive cells **(F)** in the striatum in absolute numbers for each hemisphere, related to Figures 6B–D. **(G)** Iba1 area fraction in the striatum in absolute numbers for each hemisphere, related to Supplementary Figure 4E. **p* < 0.5; ***p* < 0.01; ****p* < 0.001.

Supplementary Figure 4 **(A)** Linear regression of dopaminergic axon terminals in the striatum (from Figure 5B) vs. SA (from Figure 4C), *p* = 0.5067; *r*^2^ = 0.0335. **(B)** Linear regression of SA (from Figure 4C) vs. dendrites in SNr (from Figure 5D), *p* = 0.4127; *r*^2^ = 0.04519. **(C)** Linear regression of astroglia activation (from Figure 6B) vs. SA (from Figure 4C). *p* = 0.0609; *r*^2^ = 0.2288. **(D)** Linear regression of microglia activation (from panel **E**) vs. SA (from Figure 3C). *p* = 0.7713; *r*^2^ = 0.0062. **(E)** Microglia reaction expressed as Iba1 positive area fraction in the injected hemisphere relative to the contralateral hemisphere with the PBS-injected group set to 100%. *p* = 0.00078 for PBS vs. PFF; *p* = 0.029 for PBS vs. PFF + AS69, one-way ANOVA followed by Bonferroni *post hoc* test. Absolute values for individual hemispheres are in Supplementary Figure 3G. **p* < 0.05; ***p* < 0.01.

## Abbreviations

aSyn: alpha-synuclein
BSA: bovine serum albumin
DN: dystrophic neurite
GFAP: glial fibrillary acidic protein
Iba1: ionized calcium-binding adapter molecule 1
LB: Lewy body
LN: Lewy neurite
PBS: phosphate buffered saline solution
PD: Parkinson’s disease
PFF: pre-formed fibrils
SA: somatic accumulation
TH: tyrosine hydroxylase
WT: wild-type
DIV: day in vitro
GFAP: glial fibrillary acidic protein
Iba1: ionized calciumbinding adapter molecule 1
SNr: substantia nigra pars reticulata.
