# Supplementary material for: A β-Wrapin Targeting the N-Terminus of α-Synuclein Monomers Reduces Fibril-Induced Aggregation in Neurons
Source: Front Neurosci. 2021 Jul 13;15:696440. doi: 10.3389/fnins.2021.696440 (PMC8313869; doi:10.3389/fnins.2021.696440)
Supplement: Supplementary Figure 1 — Characterization of sonicated PFFs by atomic force microscopy (AFM). (A) AFM amplitude image of sonicated PFFs. (B) Violin plot of PFF lengths determined AFM. The white dot at 41 μm represents the median. The thick gray bar represents the interquartile range, the thin gray line represents the rest of the distribution, except for points that are determined to be outliers using a method that is a function of the interquartile range. [file Data_Sheet_1.pdf]

Supplemental Material for:

A β-wrapin targeting the N-terminus of α-synuclein monomers reduces fibril-induced aggregation in neurons

Éva M. Szegő[1*], Fabian Boß[2*], Daniel Komnig[2*], Charlott Gärtner[1], Lennart Höfs[1], Hamed Shaykhalishahi[3], Michael Wördehoff[3], Theodora Saridaki[2], Jörg B. Schulz[2,4], Wolfgang Hoyer[3], and Björn H. Falkenburger[1,2,4,5]

1) Department of Neurology, Technische Universität Dresden, Germany

2) Department of Neurology, RWTH Aachen University, Germany

3) Institut für Physikalische Biologie, Heinrich-Heine-Universität Düsseldorf, Germany and Institute of Biological Information Processing (IBI-7), Forschungszentrum Jülich GmbH, Germany

4) JARA-Institute Molecular Neuroscience and Neuroimaging, Forschungszentrum Jülich GmbH and RWTH Aachen University, Germany

5) Deutsches Zentrum für Neurodegenerative Erkrankungen, Dresden, Germany

* These authors contributed equally to this work

Address correspondence to:

Prof. Björn Falkenburger

Department of Neurology, Technische Universität Dresden

Fetscherstraße 74, 01307 Dresden, Germany

Phone: +49 351 458 2532, Fax: +49 351 458-4365, email: bfalken@ukdd.de

**Culture medium for primary neurons**		
Neurobasal A	Thermo Fisher	Cat# 10888022
B27 (2 %)	Thermo Fisher	Cat# 17504-044
Glutamax (0,5 mM)	Thermo Fisher	Cat# 35050-038
Penicillin-Streptomycin (1 %)	Thermo Fisher	Cat# 15140122

**Buffers and solutions**		
PBS, pH 7.4		
Potassium chloride (2,7 mM)		
Potassium dihydrogen phosphate (1,8 mM)		
Sodium chloride (137 mM)		
Di-Sodium hydrogen phosphate (10 mM)		
Cryopreserving Solution		
PBS		
Glycerol 30%	VWR Chemicals	Cat# 24388.295
Ethylene glycol 30%	Honeywell	Cat# 102466
TBS, pH 7.6		
Tris (50 mM)	Carl Roth	Cat# 5429.3
Sodium chloride (150 mM)		
Blocking Buffer for ICC		
TBS		
Triton X-100 (0,3 %)	Thermo Fisher	Cat# 28314
BSA (3 %)		
TBS-Tween Buffer		
TBS		
Tween 20 (0,05 %)	SERVA	Cat# 37470.01
Blocking Solution for Western Blot		
TBS		
BSA (1 %)		
Tween 20 (0,05 %)		
SDS Buffer		
Tris (75 mM)		
SDS (2%)	Carl Roth	Cat# 2326.2
Glycerol (15 %)		
EDTA (3,75 mM)	VWR Chemicals	Cat# 205-358-3
Protease Inhibitor (1:100)	Thermo Fisher	Cat# 1862209
Phosphatase Inhibitor (1:100)	Thermo Fisher	Cat# 1862495
Buffer for detergent solubility, pH 7.5		

Tris (25 mM)		
Sodium chloride (150 mM)		
EDTA (1 mM)		
Triton X-100 (1 %)		
Protease Inhibitor (1:100)		
**Equipment**		
Superdex® Increase 75 Column	GE Healthcare	Cat# GE29-1487-21
Thermomixer	Eppendorf	Model 5436
Sonicator	Biologics, Inc.	Model 300VT
Cryostat	Leica Microsystems	
Axio Imager 2 Microscope	Carl Zeiss	
Axiocam Monochrome Camera	Carl Zeiss	Axiocam 705 mono
IX81S1F Microscope	Olympus	
Luminescent Image Analyser with CCD Camera	Fujifilm	Model LAS-3000
JPK Nano Wizard II atomic force microscope	Olympus	OMCL-AC160TS
**Software**		
R (Version 2.8.0)	R Development Core Team	#RRID SCR_001905
GraphPad Prism (Version 5.01)	GraphPad	#RRID SCR_002798
ImageJ (Versions 1.47v and 1.52h)	NIH	#RRID SCR_003070
StereoInvestigator	MicroBrightfield Bioscience	#RRID SCR_002526
JPK Data Processing software	Olympus	#RRID SCR_018586
**Organisms**		
Mouse: C57BL/6J	Charles River Germany	
Mouse: C57BL/6J-Thy-A30P-α-synuclein	This paper	Kahle et al. 2000
Primary culture from mouse C57BL/6J pups	This paper	

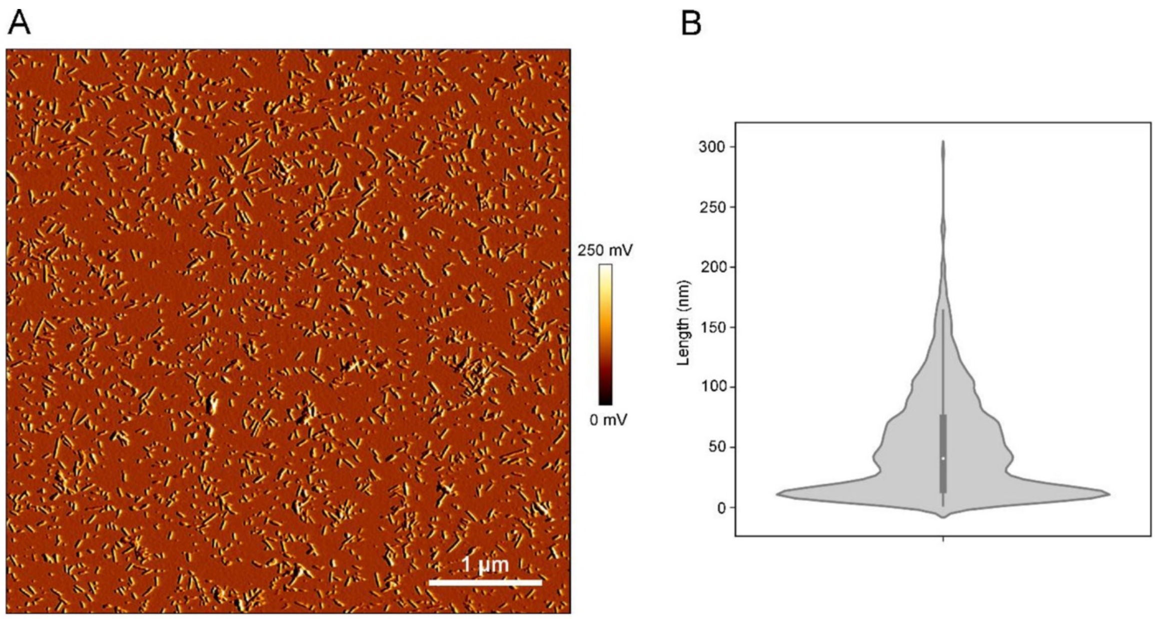

Supplemental Figure S1:

Characterization of sonicated PFFs by atomic force microscopy (AFM). (A) AFM amplitude image of sonicated PFFs. (B) Violin plot of PFF lengths determined AFM. The white dot at 41 µm represents the median. The thick gray bar represents the interquartile range, the thin gray line represents the rest of the distribution, except for points that are determined to be outliers using a method that is a function of the interquartile range.

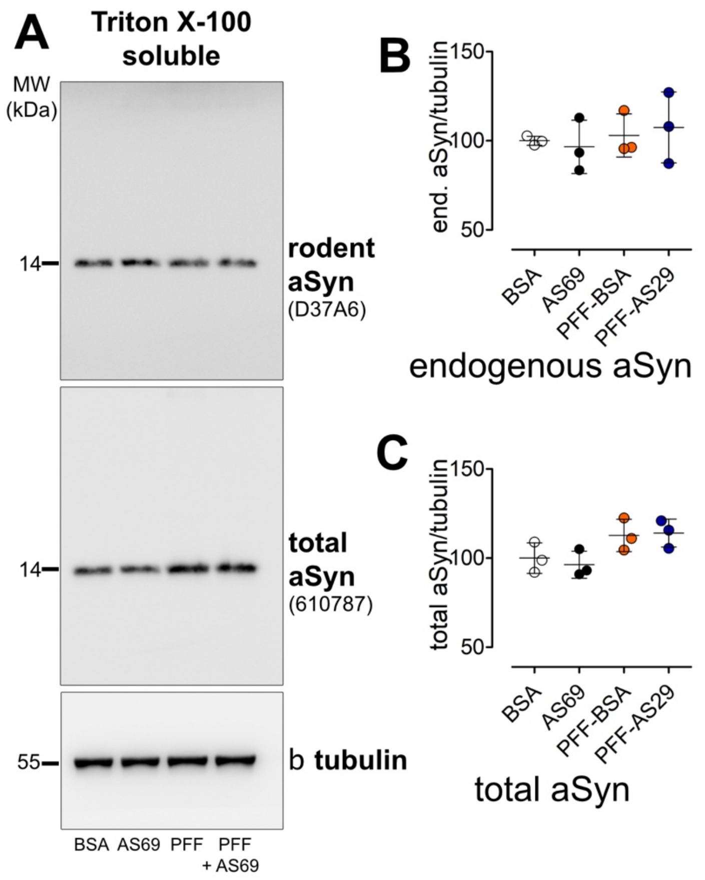

Supplemental Figure S2

(A) Representative immunoblot showing the Triton X-100 soluble fraction of primary neuron lysates obtained 10 days after PFF treatment. Blots were first incubated with an antibody detecting only rodent aSyn (D37A6, upper image), then with an antibody detecting both rodent and human aSyn (BD610787, middle image), finally βIII -tubulin was detected as loading control (lower image). (B) Quantification of the rodent aSyn signal at the 14 kDa band of n=3 independent blots as in A, showing intensity of the aSyn band relative to the βIII-tubulin band with the signal in the BSA-treated condition set to 100 %. (C) Quantification of the total aSyn signal at the 14 kDa band of n=3 independent blots as in A, showing intensity of the aSyn band relative to the βIII-tubulin band with the signal in the BSA-treated condition set to 100 %.

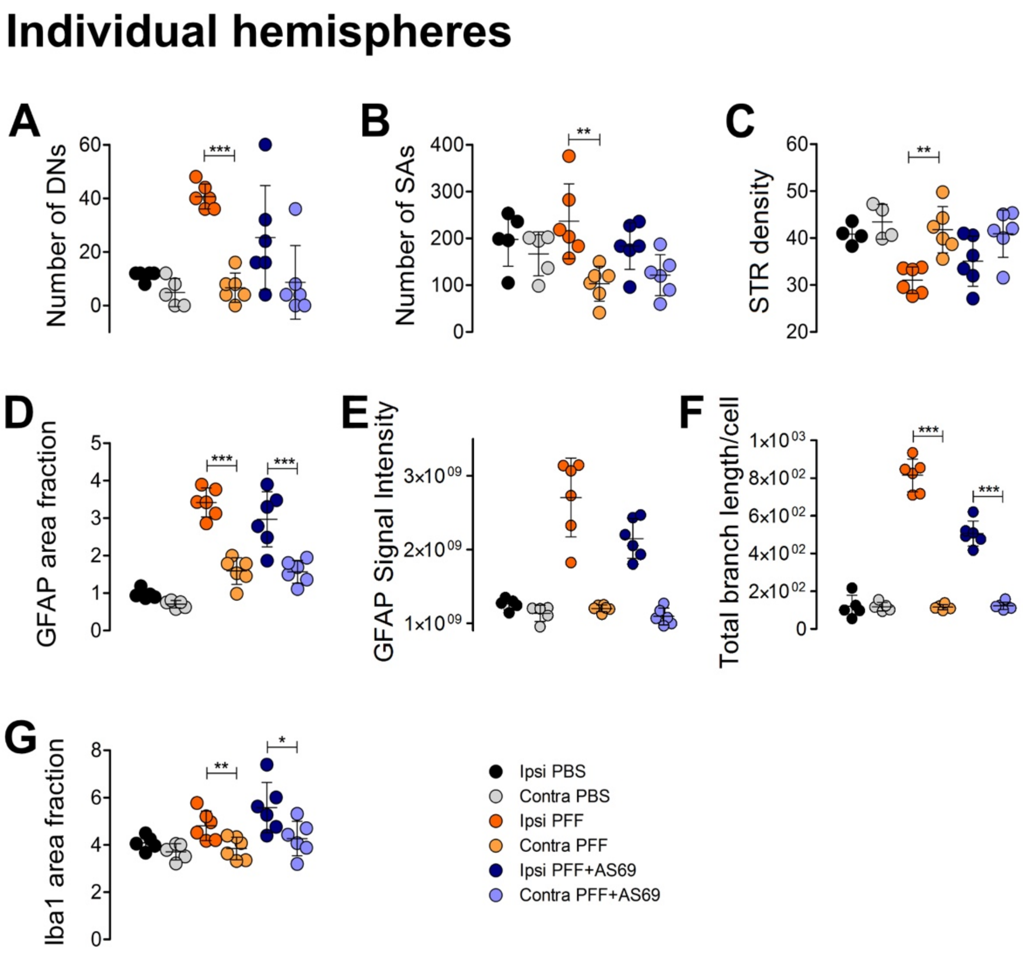

Supplemental Figure S3.

(A, B) DN and SA in the striatum in absolute numbers for each hemisphere, related to Figures 4C and D. (C) Density of dopaminergic axon terminals in the striatum in absolute numbers for each hemisphere, related to Figure 5B. (D-F) GFAP area fraction (D), GFAP staining intensity (E) and total branch length after skeletonization of GFAP positive cells (F) in the striatum in absolute numbers for each hemisphere, related to Figures 6B, c, D. (G) Iba1 area fraction in the striatum in absolute numbers for each hemisphere, related to supplemental Figure S4E.

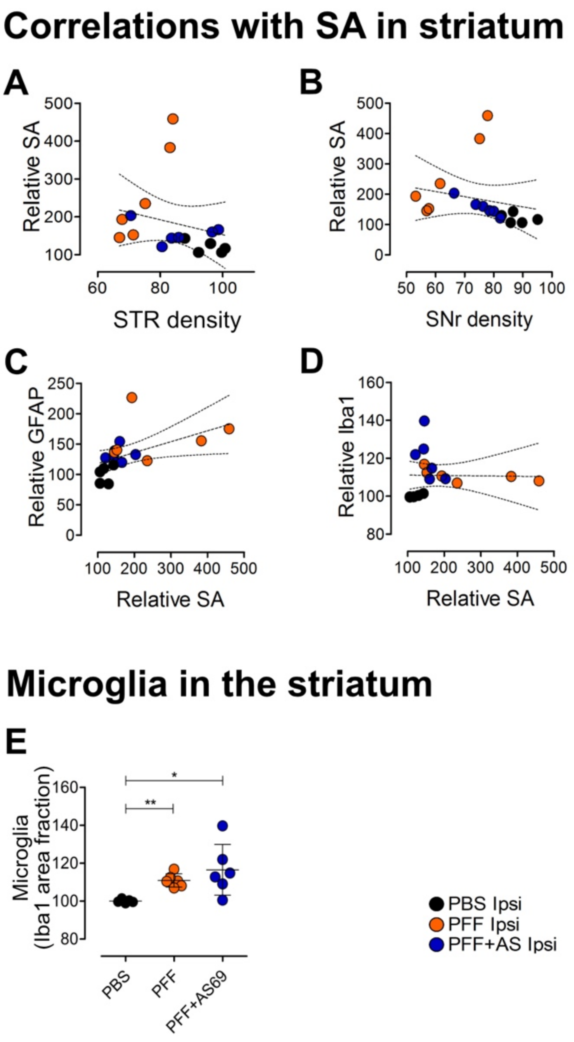

Supplemental Figure S4.

(A) Linear regression of dopaminergic axon terminals in the striatum (from Figure 5B) vs. SA (from Figure 4C), p=0,5067; r^2=0,0335. (B) Linear regression of SA (from Figure 4C) vs. dendrites in SNr (from Figure 5D), p=0,4127; r^2=0,04519. (C) Linear regression of astroglia activation (from Figure 6B) vs. SA (from Figure 4C). p=0,0609; r^2=0,2288. (D) Linear regression of microglia activation (from panel E) vs. SA (from Figure 4C). p=0,7713; r^2=0,0062. (E) Microglia reaction expressed as Iba1 positive area fraction in the injected hemisphere relative to the contralateral hemisphere with the PBS-injected group set to 100 %. p=0,00078 for PBS vs. PFF; p=0,029 for PBS vs. PFF+AS69, one-way ANOVA followed by Bonferroni post-hoc test. Absolute values for individual hemispheres are in supplemental Figure S3G.
